# Impact of smartphone addiction on health status, mental well-being, and sleep quality among medical students in Sudan

**DOI:** 10.1186/s12888-024-06377-9

**Published:** 2024-12-31

**Authors:** Mohammed Hammad Jaber Amin, Hadia Abdelbagi Alhadi, Asma Eltayeb Abdalla Mohamed, Hiba Kamal Eldin Taha Yacoub, Rabeia MohammedAhmed Hassan Khalifa, Ibrahim Abusufian Elkabashi Dafallah, Fatima Mohamed Osman Yasin, Amira Mohamed Taha, Mohammed Yousif Abdalla Adam, Majdy Osama Abualabasher

**Affiliations:** 1 Faculty of Medicine, Alzaiem Alazhari University, Khartoum, Sudan; 2 Faculty of Medicine, Omdurman Islamic University, Khartoum, Sudan; 3 Faculty of Medicine, Almughtaribeen University, Khartoum, Sudan; 4 Department of Clinical Pharmacy, University of Khartoum, Khartoum, Sudan; 5 Faculty of Medicine, Imperial University College, Khartoum, Sudan; 6 Department of Histopathology, Faculty of Medical Laboratory Science, Gadarif University, Gadarif, Sudan; 7 Faculty of Medicine, Ahfad University for women, Khartoum, Sudan; 8 Faculty of Medicine, Ibn Sina University, Khartoum, Sudan

**Keywords:** Smartphone addiction, Mental health, Sleep quality, Medical students, Sudan

## Abstract

**Background:**

Smartphone use has rapidly increased worldwide. It was estimated that worldwide use of smartphones surpassed 4.3 billion in 2023, which means 54% of the world's population now uses smartphones. However, research shows that excessive smartphone use poses health risks and decreases sleep quality, which can be detrimental for students. This study investigates the impact of problematic smartphone usage on health status, mental health, and sleep quality among medical students enrolled in Sudanese universities.

**Methods:**

A cross-sectional study was conducted from January to March 2024, targeting medical students and recent graduates from Sudanese universities through an online survey. Data were collected using validated scales for smartphone addiction, sleep quality, suboptimal health status, and mental health. Descriptive and correlation analyses were performed using SPSS software v28.0.0.

**Results:**

Out of 231 respondents (69% female, mean age 22.7), 67.6% exhibited high levels of smartphone addiction. Significant correlations were found between smartphone addiction and poor sleep quality (r = 0.462, *p* < 0.001), suboptimal health (r = 0.527, *p* < 0.001), and mental health issues (r = 0.365, *p* < 0.001). Single students had higher addiction and stress scores, while those living in Sudan showed higher suboptimal health scores.

**Conclusions:**

Problematic smartphone use (PSU) is prevalent among Sudanese medical students, negatively affecting mental health and sleep.

## Introduction

Smartphones have profoundly impacted the communication and information landscape, influencing personal and work life, while also triggering global concerns regarding overuse and potential addiction. In recent years, problematic use of smartphones popularly referred to as smartphone addiction has become a growing concern, particularly among young adults and students [1, 2]. Medical students included, are not immune to the allure of constant connectivity and the instant gratification that smartphones provide. The pervasiveness of smartphones, especially among medical students, is significant and so is the potential impact on mental well-being and sleep quality [3]. Medical students already experience considerable stress due to academic pressures. In Sudan, widespread armed conflict since April 2023 to date exacerbated the high-stress environment for medical students around the country [4]. Therefore the impact of smartphone addiction on health status, mental health and sleep quality among medical students is a topic that warrants further investigation.

Smartphone use has rapidly increased worldwide. It was estimated that worldwide use of smartphones surpassed 4.3 billion in 2023, which means 54% of the world's population now uses smartphones [5]. Mobile phones have become an integral part of youth life, offering convenience and access to a wealth of information. Smartphones stand out from ordinary mobiles due to their easy access to the web and other applications, which can be downloaded and stored.

The increased usage of smartphones among young people has led to smartphone addiction becoming a significant social issue, resulting in reported physical health problems such as musculoskeletal disorders, ocular manifestations, and an elevated risk of psychological disorders. These social problems resemble the features of behavioral addiction [6]. Diagnostic and Statistical Manual of Mental Disorders, fifth edition (DSM-5), classifies behavioral addiction as having cognitive and behavioral symptoms similar to substance-related addiction, including loss of control and withdrawal symptoms. It also acknowledges internet gaming disorder as a condition for further study [7]. In Korea, Seo et al. reported that smartphone addiction in teenagers was strongly correlated with physical symptoms, depression, anxiety, delinquency, and aggressiveness [8]. Smartphones are also reported to affect sleep outcomes. A meta-analysis of twenty studies conducted regarding the relationship of media devices and sleep outcomes showed that use of media devices was significantly associated with decreased sleep quality and duration as well as daytime sleepiness among adolescents [9].

However, it's important to critically evaluate whether excessive smartphone use truly constitutes an addiction comparable to substance abuse. The use of the term Problematic Smartphone Usage (PSU) has emerged to describe a spectrum of behavioral symptoms that may not constitute smartphone addiction [10]. Still then, problematic use of smartphones may show a significant overlap with other behavioral disorders such as the internet gaming disorder through smartphone use for internet gaming which has increased in popularity over the last few years.

This cross-sectional study aims to explore the influence of smartphone addiction on health status, mental health and sleep quality among medical students in Sudan to shed light on the potential consequences of excessive smartphone usage in this population. Understanding how smartphones impact mental health and sleep quality among medical students in Sudan can inform interventions and strategies to promote healthier technology habits and improve overall well-being in this context.

## Methods

### Study design

This is an analytical, cross sectional study about the impact of smartphone addiction on health, mental health and sleep quality among medical students in Sudan between January and March 2024. Our inclusion criteria was all medical students and recent medical school graduates enrolled in Sudanese universities, regardless of their current residence. Our study excluded those not present in various medical students online social media forums during the data collection period and those who chose not to participate in the survey. We conducted an online survey of Sudanese medical students using convenience sampling.

The total number of medical students in Sudan is unknown therefore, The sample size for an unknown population was calculated using the formula *n* = z2 P(1-P)/d2 [11]. With a 90% confidence interval, 75% response distribution and a 0.05 margin of error, a minimum sample size of 205 participants was determined to adequately represent the population.

### Data collection

We developed an online questionnaire in English language based on literature review and input from faculty members at the University of Alzaiem Alazhari (Table 1). The questionnaire consisted of 4 sections: Sociodemographic data, Suboptimal Health Status Questionnaire −25 (SHSQ-25), Sleep condition indicator, Smartphone addiction scale- Short Version (SAS-SV), and Depression, anxiety, and stress (DAS) scale. All the scales used in the study showed acceptable internal consistency and reliability in previous studies [12–15]. Participants had the option to review and change their answers using a 'back' button. We used Google Forms for data collection, which was distributed by 26 collaborators through personal and professional groups, as well as social media platforms like Facebook, WhatsApp, Twitter, and LinkedIn. We also posted study information on Sudanese social media groups for medical students and sent reminders on days 3 and 7 of the data collection period. We did not collect respondents' IP addresses to maintain anonymity and confidentiality, although Google Forms only allowed one submission per IP address. We shared information about the study with a group of collaborators to help collect data. We did not contact respondents prior to starting the study. The questionnaire included a Participant Information Sheet (PIS) and the survey link. The PIS provided details about the study's goals, protection of personal data, survey length, and the right to withdraw from the study at any time. We emphasized that participation in the survey was voluntary and there were no financial incentives.

**Table 1:** An online questionnaire in English langauage

Demographic data	Age
	Gender
	Class
	Marital status
	Residency
Sleep condition indicator	Thinking about a typical night in the last month [How long does it take you to fall asleep?]
	Thinking about a typical night in the last month [If you wake up during the night … how long does it take you to fall asleep]
	How many nights a week do you have a problem with your sleep
	How would you rate your sleep quality?
	Thinking about the past month, to what extent has sleep [Affected your mood, energy, or relationships]
	Thinking about the past month, to what extent has sleep [Affected your concentration, productivity or ability to stay awake]
	Thinking about the past month, to what extent has sleep [Troubled you in general]
	How long you have you had a problem with your sleep
Smartphone addiction scale – short version	[Missing planned work due to smartphone use]
	[Having a hard time concentrating in class, while doing assignments, or while working due to smartphone use]
	[Feeling pain in the wrists or at the back of the neck while using a smartphone]
	[Won’t be able to stand not having a smartphone]
	[Feeling impatient and fretful when I am not holding my smartphone]
	[Having my smartphone in my mind even when I am not using it]
	[I will never give up using my smartphone even when my daily life is already greatly affected by it.]
	[Constantly checking my smartphone so as not to miss conversations between other people on Twitter or Facebook]
	[Using my smartphone longer than I had intended]
	[The people around me tell me that I use my smartphone too much.]
Suboptimal Health Scale	In the preceding months, how was it that you (your)… [were exhausted without greatly increasing your physical activity?]
	In the preceding months, how was it that you (your)… [experienced fatigue that could not be substantially alleviated by rest?]
	In the preceding months, how was it that you (your)… [were lethargic when working?]
	In the preceding months, how was it that you (your)… [suffered from headaches?]
	In the preceding months, how was it that you (your)… [suffered from dizziness?]
	In the preceding months, how was it that you (your)… [eyes ached or were tired?]
	In the preceding months, how was it that you (your)… [suffered from a sore throat]
	In the preceding months, how was it that you (your)… [muscles or joints felt stiff?]
	In the preceding months, how was it that you (your)… [have pain in your shoulder/neck/waist?]
	In the preceding months, how was it that you (your)… [have a heavy feeling in your legs when walking]
	In the preceding months, how was it that you (your)… [felt out of breath while sitting still?]
	In the preceding months, how was it that you (your)… [suffered from chest congestion?]
	In the preceding months, how was it that you (your)… [were bothered by heart palpitations?]
	In the preceding months, how was it that you (your)… [Appetite was decreased?]
	In the preceding months, how was it that you (your)… [Suffered from heartburn]
	In the preceding months, how was it that you (your)… [Suffered from nausea]
	In the preceding months, how was it that you (your)… [Could not tolerate cold environment]
	In the preceding months, how was it that you (your)… [Had difficulty falling asleep]
	In the preceding months, how was it that you (your)… [Had trouble with waking up during night?]
	In the preceding months, how was it that you (your)… [Had trouble with short term memory ?]
	In the preceding months, how was it that you (your)… [Could not respond quickly?]
	In the preceding months, how was it that you (your)… [Had difficulty concentrating ?]
	In the preceding months, how was it that you (your)… [Were distracted for no reason ?]
	In the preceding months, how was it that you (your)… [Felt nervous or jittery ?]
	In the preceding months, how was it that you (your)… [Caught a cold in the past three months ?]
Depression, Anxiety and Stress Scale	[I found it hard to wind down]
	[I was aware of dryness in my mouth]
	[ I couldn’t seem to experience any positive feeling at all]
	[ I tended to overreact to situations]
	[I found it difficult to work up the initiative to do things]
	[I felt that I was using a lot of nervous energy]
	[I experienced trembling (e.g. in the hands)]
	[I was worried about situations in which I might panic and make a fool of myself]
	[I found myself getting agitated]
	[I was intolerant of anything that kept me from getting on with what I was doing]
	[ I felt down-hearted and blue]
	[I felt I was close to panic]
	[I was unable to become enthusiastic about anything]
	[I felt that I was rather touchy]
	[I felt I wasn’t worth anything as a person]
	[I was aware of the action of my heart in the absence of physical exertion (e.g. sense of heart rate increase, heart missing a beat)]
	[ I felt scared without any reason]
	[I felt that life was meaningless]
	[I experienced breathing difficulties (e.g. excessively rapid breathing, breathlessness in the absence of physical exertion)]
	[I felt that I had nothing to look forward to]
	[ I found it difficult to relax]

### Data management and statistical analysis

Responses were securely stored in Google Sheets, accessible only to the study team. The data was thoroughly cleaned and analyzed using IBM's Statistical Package for Social Sciences (SPSS) software version 28.0.0. For demographic information, continuous data was presented as mean ± SD, while categorical data was presented as numbers (percentage). To assess the normality of the data, we used the Kolmogorov–Smirnov test. For normally distributed data, we used an independent t-test to determine significant differences between demographic factors. In cases where the null hypothesis of the Kolmogorov–Smirnov test was rejected, we used the Mann–Whitney U test and Kruskal Wallis test to determine the relation of demographic variables to the smartphone addiction and the other scales. We performed a Spearman correlation testing to assess the relationships between smartphone addiction, poor sleep quality, suboptimal health status and mental health scores. For each scale the total score was used to represent smartphone addiction, sleep quality, suboptimal health status and mental health status respectively. In all correlation tests, the scales were all standardized to represent a score out of 10 to balance effects. Due to the sleep condition indicator (SCI) having a higher score for better sleep we inverted the scale to align with other scales used in the study where a higher score represents more problematic smartphone use and health conditions. A p-value of less than 0.05 was considered significant.

### Ethical approval

The study received approval from the Research Ethics Committee, Department of Community Medicine at the University of Alzaiem Alazhari, Sudan. The study adhered to the ethical standards and guidelines. Informed consent was obtained from all participants, as indicated in the data collection tool. To ensure comprehensive and accurate reporting of the study findings, we followed the Checklist for Reporting Results of Internet E-Surveys (CHERRIES) [16]. We confirm that all methods were conducted in accordance with relevant research ethics guidelines and regulations. Participants provided informed consent at the start of the online survey before completing the questionnaire.

### Clinical trial number

Not applicable.

## Results

**Fig. 1:**
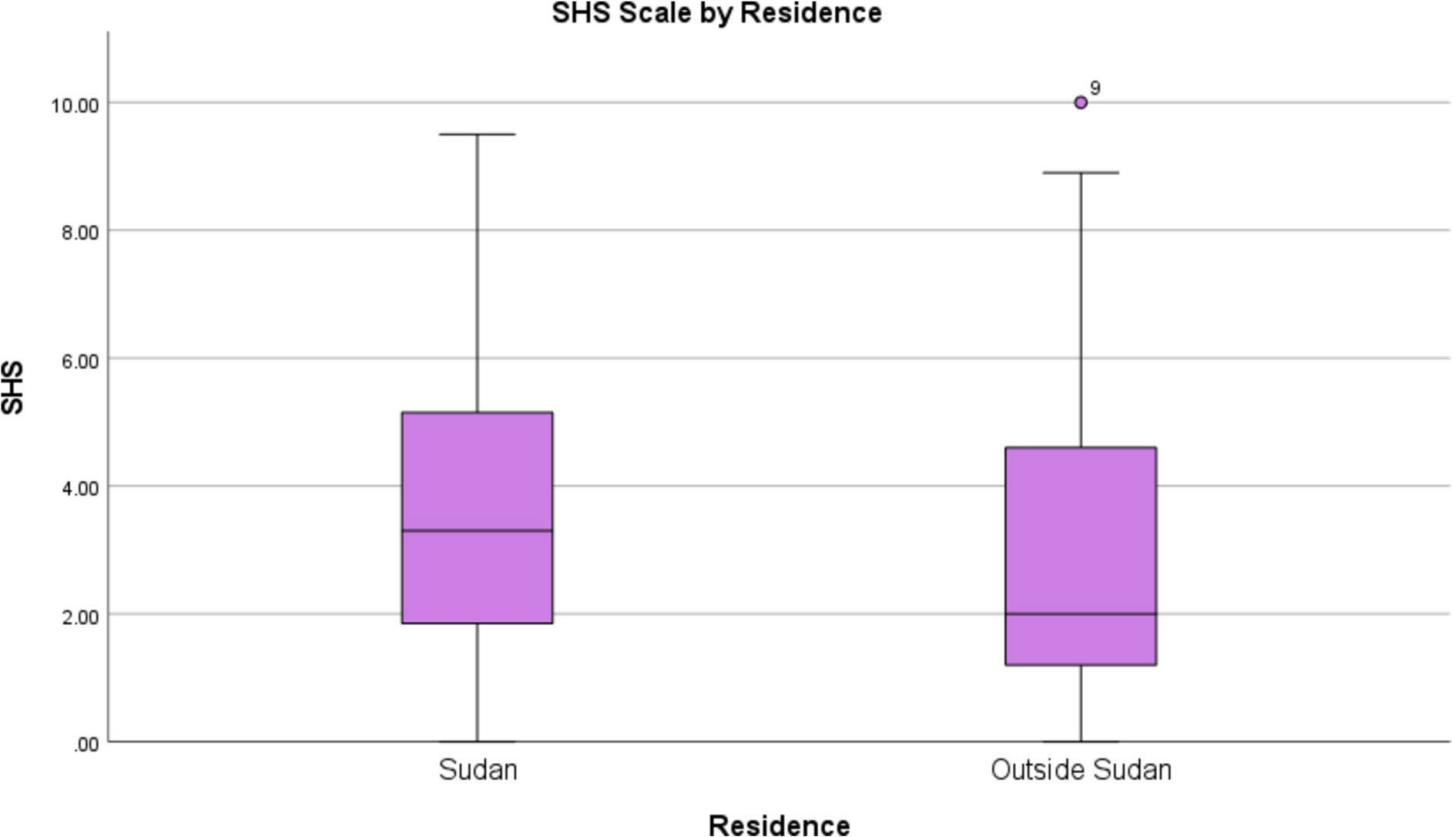
Represent association between smartphone addiction scale and marital status

A total of 231 questionnaires were collected with a response rate of 100%. Of all the participants, 147 (69.0%) were female and 66 (31.0%) were male. The mean age of the participants was 22.7 years (SD ± 2.34). Age was classified into categories as follows: 17- 20 years, 27 (12.7%); 21–23 years, 122 (57.3%); and more than 23 years, 64 (30%). Among these, 7 (3.3%) were in the first level of university, followed distantly by 19 (8.9%) in the second level, 54 (25.4%) in the third level, 66 (31.0%) in the fourth level, 45 (21.1%) in the fifth level, 16 (7.4%) in the sixth level, and 6 (2.8%) had graduated from university. The study revealed that 17 (8.0%) were single, while the majority, 196 (92%), were married. Moreover, only 99 (46.55%) of the participants lived inside Sudan, while 114 (53.5%) lived outside Sudan (Table 2 and Fig. 1).

**Table 2:** Participants’ demographic characteristics (*n* = 231)

Variables	Frequency	Percent %
Gender		
Female	147	69
Male	66	31
Age		
17–20	27	12.7
21–23	122	57.3
Above 23	64	30
Martial Status		
Single	196	92
Married	17	8
Academic Year		
1st	7	3.3
2nd	19	8.9
3rd	54	25.4
4th	66	31
5th	45	21.1
6th	16	7.4
Recently Graduated	6	2.8
Residence	99	46.5
In Sudan		
Outside Sudan	114	53.5

The Spearman correlation analysis revealed that the SCI showed a positive correlation with SAS-SV (r = 0.462, *p* < 0.001). SCI was positively correlated with SHSQ-25 (r = 0.549, *p* < 0.001) and DAS (r = 0.470, *p* < 0.001). The SAS-SV also demonstrated significant correlations with SHSQ-25 (r = 0.527, *p* < 0.001) and DAS (r = 0.365, *p* < 0.001). Furthermore, SHSQ-25 and DAS were strongly correlated (r = 0.624, *p* < 0.001) (Table 3).

**Table 3:** Spearman rank test for SAS, SCI, SHS and DAS scales (*n* = 213)

		SCI	SAS	SHS	DAS
SCI	Correlation Coefficient		.462	.549	.470
	*p-*value		< .001	< .001	< .001
SAS	Correlation Coefficient	.462		.527	.365
	*p-*value	< .001		< .001	< .001
SHS	Correlation Coefficient	.549	.527		.624
	*p-*value	< .001	< .001		< .001
DAS	Correlation Coefficient	.470	.365	.624	
	*p-*value	< .001	< .001	< .001	

Mann–Whitney U tests were conducted to compare smartphone addiction scores between genders (Table 4). No significant differences were found across the SAS-SV (U = 4867.000, *p* = 0.969), SCI (U = 4952.500, *p* = 0.807), SHSQ-25 (U = 4541.000, *p* = 0.456), and DAS (U = 4877.500, *p* = 0.949) scales between female and male participants Table 4.

**Table 4:** Mann Whitney U test for SAS, SCI, SHS and DAS against gender

	N (Females)	N (Male)	Mann–Whitney U	Z Score	*p-*value
SAS	147	66	4867.000	.038	.969
SCI	147	66	4952.500	.244	.807
SHS	147	66	4541.000	-.745	.456
DAS	147	66	4877.500	.064	.949

### Mann Whitney U test for SAS-SV, SCI, SHSQ-25 and DAS against gender

Marital status was associated with significant differences in smartphone addiction scores, as shown in Table 5. Single participants exhibited higher scores on the SAS-SV (U = 2516.500, *p* < 0.001), SCI (U = 2554.000, *p* < 0.001), SHSQ-25 (U = 3536.000, *p* < 0.001), and DAS (U = 2335.000, *p* = 0.006) scales compared to married participants.

**Table 5:** Mann Whitney U test for SAS, SCI, SHS and DAS against martial status

	N (Married)	N (Single)	Mann–Whitney U	Z Score	*p-*value
SAS	17	196	2516.500	3.491	< .001
SCI	17	196	2554.000	3.648	< .001
SHS	17	196	3536.000	3.570	< .001
DAS	17	196	2335.000	2.746	.006

The impact of residence on smartphone addiction and wellness was evaluated as shown in Table 6. Only the SHSQ-25 scores showed a significant difference, with participants residing outside Sudan scoring higher (U = 4508.500, *p* = 0.011), while no significant differences were detected for SAS-SV (U = 5062.000, *p* = 0.195), SCI (U = 6249.500, *p* = 0.176), and DAS (U = 5358.000, *p* = 0.525).

**Table 6:** Mann Whitney U test for SAS, SCI, SHS and DAS against residence

	N (In Sudan)	N (Outside Sudan)	Mann–Whitney U	Z Score	*p-*value
SAS	99	114	5062.000	−1.296	.195
SCI	99	114	6249.500	1.354	.176
SHS	99	114	4508.500	−2.529	.011
DAS	99	114	5358.000	-.636	.525

The Kruskal–Wallis test was employed to analyze differences in smartphone addiction across academic years (Table 7). The results indicated no significant differences in SAS-SV (χ^2^ = 7.600, *p* = 0.269), SCI (χ^2^ = 3.896, *p* = 0.691), SHSQ-25 (χ^2^ = 5.510, *p* = 0.480), or DAS (χ^2^ = 4.566, *p* = 0.601) scores among the different academic years.

**Table 7:** Kruskal Wallis test for SAS, SCI, SHS and DAS across class years

Variable	N (total)	Chi-square	Df	*p-*value
SAS	213	7.600	6	.269
SCI	213	3.896	6	.691
SHS	213	5.510	6	.480
DAS	213	4.566	6	.601

## Discussion

### General

To our knowledge, this study is the first investigation into smartphone addiction among medical students in Sudan.

### Age

Our study revealed no significant correlation between problematic smartphone use and year of study ( *p* = 0.269). It is likely that the reason for this is a similar occupation (student) for all the participants.

A study previously conducted in India reported a 37% smartphone addiction rate among adolescents, more than that of medical students in a similar study [17]. A study also found a higher likelihood of addiction among those who started using mobile phones at a younger age [18]. Our results suggest that neither age nor academic stage necessarily correlate with problematic smartphone use. Rather, smartphones have become an integral part of students' lives. This is likely due to their reliance on online medical references in our digital age [19].

### Gender

Our study also found no significant differences in smartphone addiction by gender. This sample challenges some existing literature that typically finds higher addiction levels among females for example study that also observed a higher prevalence of smartphone addiction among females [20]. The results reported in our study might be attributed to changing societal norms and the increasing prevalence of smartphones among all demographics. Recent studies suggest that as smartphone usage becomes more universal, the gap between genders in terms of addiction may diminish [21].

Moreover, the types of activities engaged in on smartphones may differ by gender, with males often gravitating towards gaming and females towards social media [22, 23]. This divergence in usage patterns may impact overall addiction levels, suggesting that future research should explore the specific applications and activities that contribute to addiction, rather than focusing solely on device usage.

### DAS

Our study showed a significant correlation between DAS and SAS-SV scales (*p* < 0.001). Furthermore, SHSQ-25 and DAS were strongly correlated (*p* < 0.001) highlighting the well researched fact that physical health affects mental health scores (Table 3).

A Jordanian study supports this, showing a significant positive correlation between smartphone addiction, depression, and anxiety among medical students [24]. In Iran, a study involving university students found that smartphone addiction can negatively impact both the physical and mental aspects of students' quality of life [25]. In a prior study involving fifth-year students, it was found that 97.2% of medical students experienced anxiety during the OSCE exam [26]. As smartphones serve as multifunctional tools for studying, socializing, and entertainment, their pervasive presence may blur the lines between healthy use and problematic use [10].

### SCI

Our study showed a significant correlation of SCI with SAS-SV (*p* < 0.001), possibly due to disrupted sleep patterns caused by excessive screen time, especially before bed. Smartphone activities can stimulate the mind and make it difficult to fall asleep and maintain restful sleep. This supports earlier findings that suggest smartphone addiction disrupts sleep [27]. A study in South India found poor sleep quality in 77 medical students who used smartphones [28]. Another study suggests a link between sleep quality and academic performance, with better grades achieved by those who go to bed between 10:00 and 11:00 PM [29]. Research has also shown that undergraduate students at risk of smartphone addiction are less likely to achieve high cumulative GPAs [30, 31].

### Residence

Medical students in Sudan showed a relatively high level of smartphone addiction, surpassing similar studies from other countries [32, 33]. A study examined smartphone addiction among university students in Sudan, Jordan, Saudi Arabia, and Yemen and found that Sudanese students had a higher tendency towards smartphone addiction compared to Yemeni students. Jordanian and Saudi students had a higher prevalence of smartphone addiction than Sudanese students [20]. This aligns with a global analysis performed in 24 countries which reports a higher prevalence of smartphone addiction in countries like Saudi Arabia and China while less use is reported in countries like France and Germany [34]. Notably Saudi Arabia and Gulf countries are a major destination for Sudanese expatriates and their families due to work opportunities. This resulted in many students who left Sudan due to the war after April 2023 to join their families and relatives in the Gulf region and thus we notice higher levels of smartphone addiction among students outside Sudan than those in Sudan, even if this difference is not significant. This finding aligns with the concept of digital divide, where disparities in access to technology can lead to varying levels of engagement and addiction [35].

**Fig. 2:**
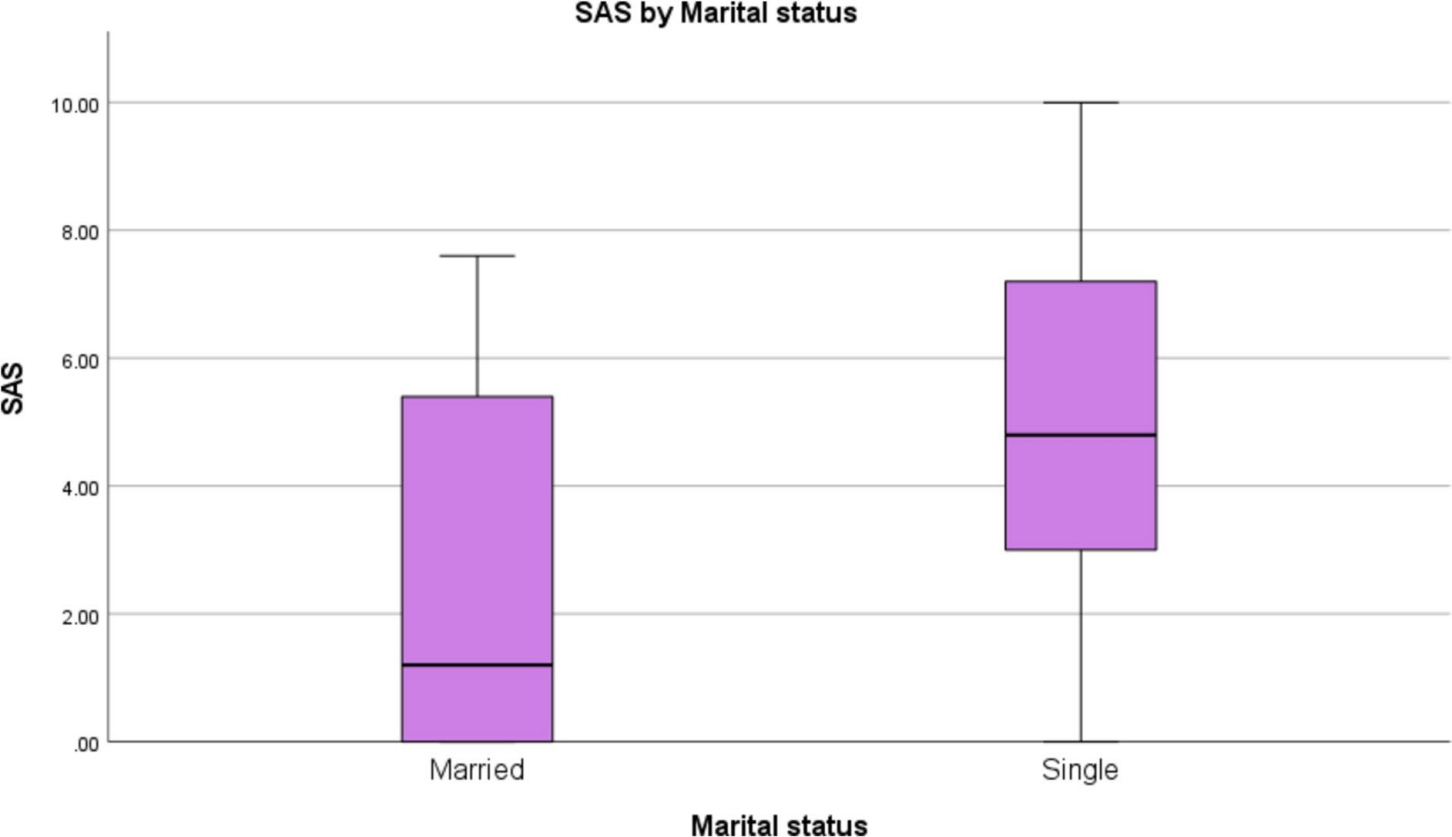
Represent association between smartphone addiction scale and marital status

The significant difference in SHSQ-25 scores between students living in Sudan and those residing outside the country highlights the influence of residence on health status and smartphone usage (Table 4 and Fig. 2). It is possible high levels of SHSQ-25 observed in Sudanese medical students in this study may be due to the ongoing armed conflict in Sudan since April 2023. Previous research conducted in Ukraine during wartime also found a high prevalence of severe anxiety and manifestations among university students in war-affected regions [36]. Interestingly this difference in SHSQ-25 did not affect the DAS scales.

### Martial status

Our study showed that married couples are less likely to have problematic sleep usage, sleep disturbance, suboptimal health status and mental illness. The significant association between marital status and smartphone addiction (*p* < 0.001), sleep condition (*p* < 0.001), health status (*p* < 0.001), depression, anxiety and stress (*p* = 0.006) raises important questions about the psychosocial dynamics influencing technology use among young adults.

Single individuals may utilize smartphones as a primary means of social connection, particularly in environments where face-to-face interaction is limited. This reliance on digital communication could serve as a coping mechanism for loneliness or social anxiety, a phenomenon documented in previous research [37].

Furthermore, the developmental stage of young adulthood is characterized by exploration and identity formation. For single students, smartphones may represent not only a tool for social interaction but also a platform for self-presentation and validation. Studies indicate that social media engagement is linked with self-esteem and peer acceptance [38]. Consequently, students may feel compelled to maintain an active online presence, leading to excessive smartphone usage and potential addiction.

The higher prevalence of smartphone addiction among single medical students compared to their married counterparts could be influenced by several factors. Single individuals often have different social dynamics and potentially more free time, which may lead to increased smartphone usage for entertainment, social interaction, or other non-academic purposes. Married medical students may experience a different set of responsibilities and time constraints, potentially reducing their leisure time for smartphone-related activities. The presence of a spouse and family obligations might also contribute to a more balanced approach to technology use among married students. Furthermore, social support within a marital relationship could offer alternative sources of companionship and entertainment, reducing the reliance on smartphones for these needs. However, a prior study indicated that in the clinical years, 41% to 59% of medical students utilized smartphones for lecture notes, medical videos, electronic textbooks, and medical research [19]. Therefore, the strong association of smartphone addiction with a wide variety of influences warrant further investigation into contextual factors. This research supports existing theories on smartphone addiction and mental health concerning medical students [39].

## Conclusion

In conclusion, our study sheds light on the significant prevalence of smartphone addiction among medical students in Sudan, with 67.6% of participants exhibiting high levels of addiction. This addiction has profound implications for health, particularly in terms of negatively impacting sleep patterns. The findings underscore the need for tailored interventions and awareness programs targeting smartphone addiction, considering demographic factors such as age, gender, and marital status. Additionally, our study emphasizes the importance of further research to explore contextual factors contributing to smartphone addiction among medical students and to develop effective strategies for intervention and prevention.

### Limitations

While the online survey provided valuable insights into the views of Sudanese medical students on smartphone addiction during conflict, it is important to acknowledge certain limitations. The study relied on online data collection and non-probability sampling methods, which may have introduced selection bias. This is because participation was dependent on internet access and familiarity with online surveys, potentially excluding individuals without such access. Additionally, the survey's self-report format increases the risk of response bias, as participants could have provided socially desirable responses or misrepresented their experiences. Furthermore, the study's focus on quantitative data restricts the depth of qualitative insights that could have been obtained.

### Recommendations

One of the most simple and smart techniques to avoid phone addiction is using apps that restrict daily usage and placing the charging station outside bedrooms to reduce late-night phone use. Practicing mindfulness is important. Also, one should consider engaging in consistent activities to cope with psychological stress, seek support from family, friends, and additional therapy or counseling to manage mental health, especially symptoms of depression. Students should also strive to preserve their health through adequate hydration, nutrition, hygiene, exercise and health seeking behavior. Educational institutions should consider integrating digital literacy and well-being into their curricula.

## Data Availability

The datasets used and analyzed during the current study are available and can be accessed through the link: https://osf.io/h9kv7/?view_only=1ce5e53ad07445b193e09dad8d889075. The authors declare that they have no competing interests [40].
